# Efficacy of allergen-specific immunotherapy for allergic rhinitis: a meta-analysis

**DOI:** 10.3389/fmed.2026.1759079

**Published:** 2026-03-24

**Authors:** Dong Wang, Didi Song, Xiaoqian Xu

**Affiliations:** Department of ENT, The 902nd Hospital, Joint Logistic Support Force of the PLA, Bengbu, Anhui, China

**Keywords:** allergen-specific immunotherapy, allergic rhinitis, meta-analysis, subcutaneous immunotherapy, sublingual immunotherapy

## Abstract

**Objective:**

This meta-analysis aimed to systematically evaluate and compare treatment adherence (measured by treatment discontinuation rate) and safety (measured by adverse event rate) between subcutaneous immunotherapy (SCIT) and sublingual immunotherapy (SLIT) in patients with allergic rhinitis (AR), providing evidence-based guidance for clinical decision-making.

**Methods:**

A comprehensive literature search was conducted in PubMed, MEDLINE, Web of Science, Cochrane Library, and EMBASE databases from inception to August 2025 to identify relevant studies (specifically randomized controlled trials [RCTs]) comparing SCIT and SLIT for treating AR. Two independent researchers performed study selection and data extraction. The methodological quality of RCTs was evaluated using the Cochrane RoB-2 tool. Statistical analyses were performed using RevMan 5.3 and STATA 18.0. Dichotomous data were expressed as odds ratios (ORs), and continuous data were expressed as mean differences (MDs), both with 95% confidence intervals (CIs). Heterogeneity was assessed using the I^2^ statistic. Publication bias and robustness of results were examined using Egger’s test and sensitivity analyses.

**Results:**

This meta-analysis showed that there was no statistically significant difference in the treatment discontinuation rate between SCIT and SLIT in the treatment of allergic rhinitis (combined log OR = 0.30, 95% CI [−0.79, 1.39], *p* = 0.59); the incidence of adverse events in SCIT was significantly higher than that in SLIT (pooled log OR = 0.60, 95% CI [0.05, 1.15], *p* = 0.03), with a statistically significant difference. In clinical practice, the treatment plan can be selected based on patients’ preferences for treatment methods, their need for convenience, and the conditions for monitoring. Future large-sample long-term studies are needed for further validation of the findings.

**Conclusion:**

Both SCIT and SLIT exhibited comparable treatment adherence (discontinuation rates) in patients with allergic rhinitis; however, the incidence of adverse events in SCIT was significantly higher than that in SLIT, and SLIT had more advantages in terms of safety. Clinical decisions should consider individual patient factors, including treatment convenience and monitoring conditions.

**Systematic review registration:**

https://www.crd.york.ac.uk/prospero/display_record.php, identifier CRD420261306427.

## Introduction

Allergic rhinitis (AR) is one of the most prevalent allergic diseases worldwide. It is characterized by typical symptoms such as nasal congestion, rhinorrhea, nasal itching, and paroxysmal sneezing. It not only significantly impairs patients’ daily work, academic performance, and sleep quality but may also lead to complications such as asthma and sinusitis with long-term recurrent episodes, posing a substantial threat to physical and mental health (1). Epidemiological data indicate that the global prevalence of AR has reached between 10 and 30%, and with increasing urbanization and environmental changes, its incidence continues to rise annually, imposing a heavy burden on public health systems (2).

Currently, the clinical management of AR primarily focuses on symptomatic control, including pharmacotherapies such as antihistamines and intranasal corticosteroids. However, these treatments only alleviate symptoms without fundamentally altering the immune response to allergens, leading to symptom recurrence upon discontinuation (3). In contrast, allergen-specific immunotherapy (AIT), the only disease-modifying treatment capable of altering the natural course of AR, induces immune tolerance by gradually increasing allergen exposure, leading to long-term symptom control. AIT has become a crucial therapeutic option for individuals with moderate-to-severe AR (4).

AIT primarily includes two administration routes: subcutaneous immunotherapy (SCIT) and sublingual immunotherapy (SLIT). SCIT, the conventional approach, involves regular subcutaneous injections of allergen extracts, and its efficacy is well established in clinical practice (5). SLIT, a newer modality, induces immune tolerance through sublingual administration of allergens. This method offers advantages in terms of convenience and ease of self-administration, which can potentially enhance patient adherence (6). In recent years, as both therapies have gained widespread clinical use, an increasing number of studies have compared their efficacy and safety; however, the findings remain controversial. Some studies suggest that SCIT provides superior symptom improvement (7), while others highlight the advantages of SLIT in terms of safety and adherence (8, 9). However, individual studies often have limited sample sizes and heterogeneous populations, making it difficult to draw definitive conclusions. Existing studies on this topic often report mixed outcomes (e.g., mixing symptom scores with adherence) or include observational studies, leading to inconsistent conclusions regarding core clinical decision indicators (particularly adherence and safety). Moreover, high-quality RCTs that directly compare SCIT and SLIT for these two core outcomes are scarce. Thus, conducting this meta-analysis is necessary to systematically integrate the currently available high-quality evidence, even if the number of studies is limited, and to clarify the inconsistent conclusions from individual small-sample studies. We will quantitatively assess differences in treatment discontinuation and adverse event rates between SCIT and SLIT to fill the research gap and provide targeted evidence for clinical therapy selection.

Therefore, this study aims to systematically evaluate and conduct a meta-analysis of global studies comparing SCIT and SLIT for AR. We will quantitatively assess their clinical value across multiple dimensions, including treatment adherence and safety, to provide high-quality evidence-based guidance to clinicians in selecting optimal AIT strategies tailored to individual patients.

## Materials and methods

### Inclusion and exclusion criteria

The inclusion criteria are as follows: (1) study population: patients diagnosed with AR based on clinical symptoms, signs, and allergen testing (e.g., skin prick test and serum-specific IgE). No restrictions on age, sex, or disease duration, but studies must clearly differentiate between SCIT and SLIT groups. (2) Interventions: Studies must compare SCIT (specifying allergen type, dosage, injection frequency, and treatment duration) and SLIT (detailing allergen type, administration regimen, dosage, and treatment period), with comparable baseline characteristics between the groups. (3) Study design: randomized controlled trials (RCTs) with clear descriptions of grouping methods, data collection procedures, and quality control measures. (4) Outcomes: Studies must report at least one of the following extractable outcomes: (1) Adherence measured as the treatment discontinuation rate (number of discontinuations/total number of participants) and (2) Safety, assessed through the adverse event rate (number of events/total number of participants). (5) Data must allow for the derivation of effect sizes (mean differences [MDs], odds ratios [ORs]) with 95% confidence intervals (95% CI).

The exclusion criteria are as follows: (1) animal studies, reviews, conference abstracts, and case reports and (2) studies with incomplete or unextractable data.

### Outcome definitions and assessment criteria

(1) Treatment discontinuation rate: The percentage of patients who stop treatment prematurely due to adverse events, lack of efficacy, or personal preference, which reflects adherence. (2) Adverse event rate: The percentage of patients who experience local (e.g., injection-site reactions and sublingual irritation) or systemic (e.g., dizziness, rash, and dyspnea) reactions, which are assessed to evaluate safety.

The primary outcome indicators specified in this study are the treatment discontinuation rate and adverse event rate. The extraction and analysis of all outcome indicators are strictly conducted in accordance with the prespecified standards.

### Search strategy

(1) The databases utilized are PubMed, MEDLINE, Web of Science, Cochrane Library, and EMBASE.
(2) The keywords employed in this search are “Allergen Immunotherapy,” “AIT,” “Sublingual Immunotherapy,” “SLIT,” “Subcutaneous Immunotherapy,” “SCIT,” “Allergic Rhinitis,” “Treatment Outcome,” “Efficacy,” “Effectiveness,” “randomized controlled trial”, and “RCT.”
Example: (“Allergen Immunotherapy” OR “AIT” OR “Sublingual Immunotherapy” OR “SLIT” OR “Subcutaneous Immunotherapy” OR “SCIT”) AND “Allergic Rhinitis” AND (“Treatment Outcome” OR “Efficacy” OR “Effectiveness”) AND (“randomized controlled trial” OR “RCT”).
(3) The timeframe for the search extends from the inception of each database to August 2025.

Our study was registered on the International Prospective Register of Systematic Reviews under the registration number CRD420261306427.

### Study selection and data extraction

Two independent reviewers screened the studies and extracted the data. Any discrepancies were resolved through discussion or third-party arbitration. The extracted data included study characteristics (author, year, country, and design), patient demographics (age, sex, allergen type, and disease duration), intervention details (AIT type, allergen, dosage, and treatment duration), outcome measures (discontinuation/adverse event rates), and key time points and follow-up duration.

### Quality assessment

RCTs were evaluated using the Cochrane Risk of Bias Tool (Cochrane RoB-2 tool) This tool was used to evaluate random sequence generation (selection bias), allocation concealment (selection bias), blinding of participants/personnel (performance bias), blinding of outcome assessment (detection bias), incomplete outcome data (attrition bias), and selective reporting (reporting bias). The Cochrane RoB-2 tool assessed randomization, blinding, data completeness, and reporting bias, classifying studies as having “low,” “high,” or “unclear” risk.

### Statistical analysis

Meta-analysis was performed using RevMan 5.3 and STATA 18.0. Dichotomous data were expressed as ORs, and continuous data were expressed as MDs (both with 95% CIs). The fixed-effects model was used if heterogeneity was low (I^2^ < 25%, *p* > 0.1); otherwise, the random-effects model was applied. Funnel plots and sensitivity analyses assessed publication bias and the robustness of results.

## Results

**Figure 1:**
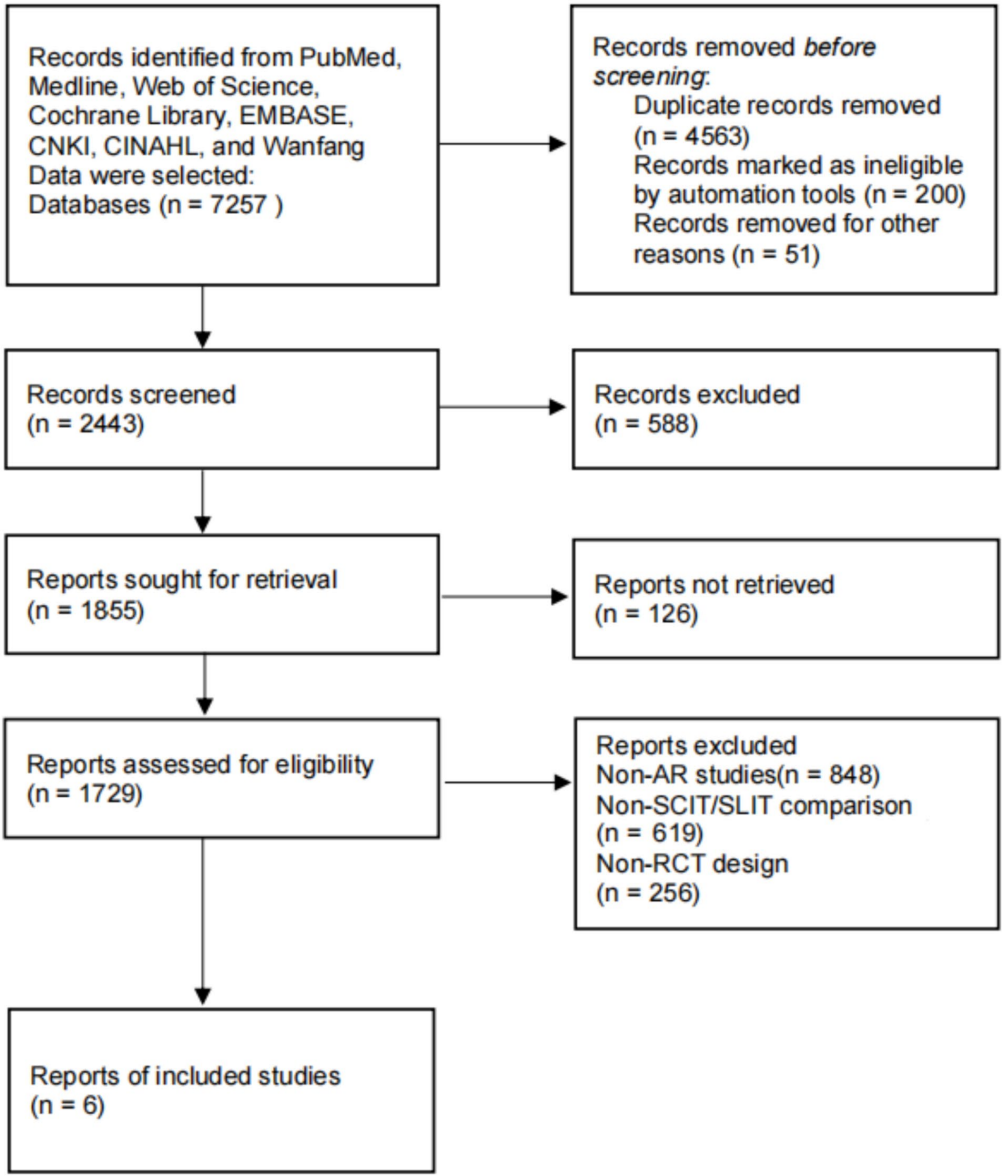
Literature screening flowchart.

Database searches identified 7,257 records. After deduplication, 2,754 full-text articles were screened, with 6 studies meeting the inclusion criteria (Figure 1).

### Characteristics of included studies

A total of 6 studies involving 588 participants were included, all of which were RCTs. The basic characteristics of the included studies are presented in Table 1.

**Table 1:** Basic characteristics of the included studies.

Reference	Sample size (SCIT/SLIT)		Allergen type and dose	Definition of treatment discontinuation	Adverse event severity classification	Outcome measures	Study type
	SCIT group	SLIT group					
Liu 2020 (10)	80	160	2 years	Allergen type: House dust mite (Der f) SCIT: Alutard (Der p extract, 100000 SQ-U/mL), NHD (Der p/Der f 50/50, 5,000 TU/mL) SLIT: Der f drops (No. 1–4, concentration 1–333 μg/mL)	Discontinuation of treatment in advance (due to adverse events, insufficient efficacy, or personal willingness); children who completed 2-year treatment were defined as adherent	Treatment discontinuation rate, adverse event rate	RCT
Wang 2017 (14)	34	34	1 year	Allergen type: House dust mite (Der p/Der f) SCIT: Mixed allergen extract (concentration 1:10000–1:100, dose 0.1–1.0 mL) SLIT: Single Der f drops (No. 1–5, concentration 1–1,000 μg/mL)	Discontinuation of treatment in advance (failure to complete 1-year treatment for any reason)	Treatment discontinuation rate	RCT
Dranitsaris 2014 (11)	73	80	1 pollen season	Allergen type: 5 grass pollens (*Dactylis glomerata*, *Poa pratensis*, etc.) SCIT: Standardized extract (dose increased from 0.2–0.8 mL during the induction phase, monthly during the maintenance phase) SLIT (Oralair™): 300 IR/day, 4 months preseason + 2 months in-season	Discontinuation due to intolerable adverse events	Treatment discontinuation rate, adverse event rate	RCT
Scadding 2017 (12)	36	36	3 years	Allergen type: *Phleum pratense* pollen SCIT: Alutard SQ (20 μg Phleum p 5 per dose, monthly during the maintenance phase) SLIT: Grazax™ (15 μg Phleum p 5 per day)	Failure to complete 3-year treatment or early withdrawal; those who completed were defined as adherent	Treatment discontinuation rate	RCT
Yukselen 2011 (13)	10	11	1 year	Allergen type: House dust mite (Der p/Der f 50/50) SCIT: Novo-Helisen Depot (50–5,000 TU/mL, weekly during the induction phase, every 4 weeks during the maintenance phase) SLIT: Novo-Helisen Oral (10–1,000 TU/mL, 12-week induction phase, 3 times a week during the maintenance phase)	Discontinuation due to adverse events, insufficient efficacy, or loss to follow-up	Treatment discontinuation rate, adverse event rate	RCT
Mauro 2007 (20)	19	15	1 pollen season	Allergen type: Mixed birch, alder, and hazel pollen extract SCIT: Phostal^®^ (8 IR per dose, every 3 weeks during the maintenance phase) SLIT: Staloral^®^ (100 IR per day, 11-day induction phase)	Failure to complete treatment during pollen season (early withdrawal for any reason)	Adverse event rate	RCT

### Quality assessment

**Figure 2:**
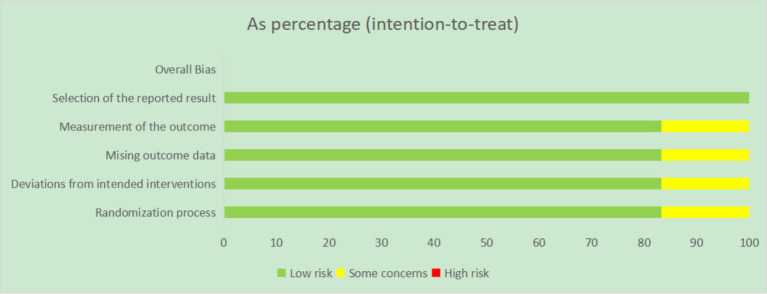
Risk of bias distribution graph for the included studies.

The results showed an unclear risk of bias in domains such as random sequence generation and allocation concealment; however, no studies had definitive high-risk biases. Overall, the bias distribution was balanced (Figure 2).

### Meta-analysis

#### Treatment discontinuation rate

**Figure 3:**
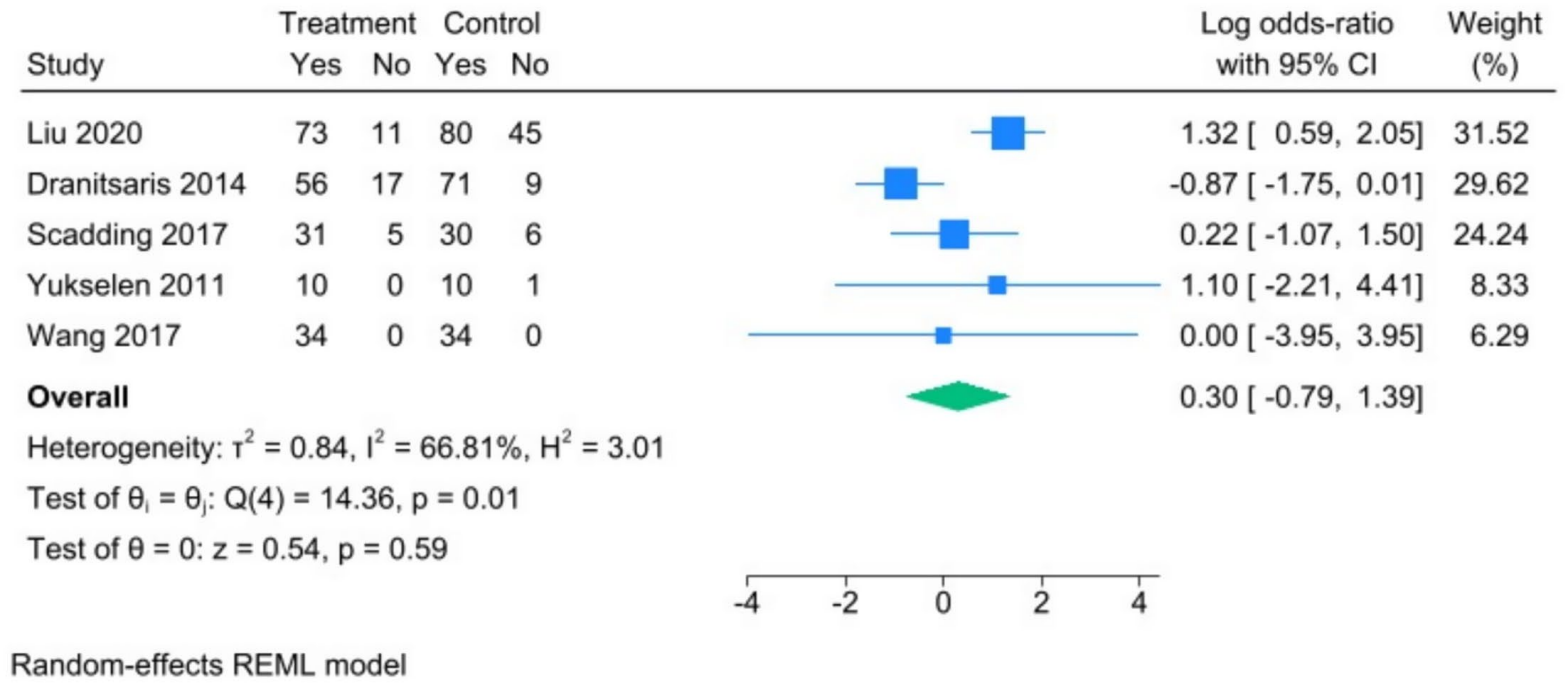
Forest plot of the treatment discontinuation rate.

The forest plot of treatment discontinuation rates showed that five studies investigated the correlation between SCIT and SLIT treatment discontinuation rates in allergic rhinitis. Heterogeneity assessment via Q-test and I^2^ statistic showed significant heterogeneity among the included studies (Chi^2^ = 14.36, df = 4, *p* = 0.01, I^2^ = 66.81%). Therefore, a random-effects model was adopted to synthesize the data. The meta-analysis results indicated that there was no statistically significant difference in the risk of treatment discontinuation between the SCIT and SLIT groups (combined log OR = 0.30, 95% CI [−0.79, 1.39], Z = 0.54, *p* = 0.59). This result indicates no significant difference in compliance between the SCIT and SLIT groups, as measured by the discontinuation rate, in the treatment of allergic rhinitis (Figure 3).

#### Adverse reaction incidence rate

**Figure 4:**
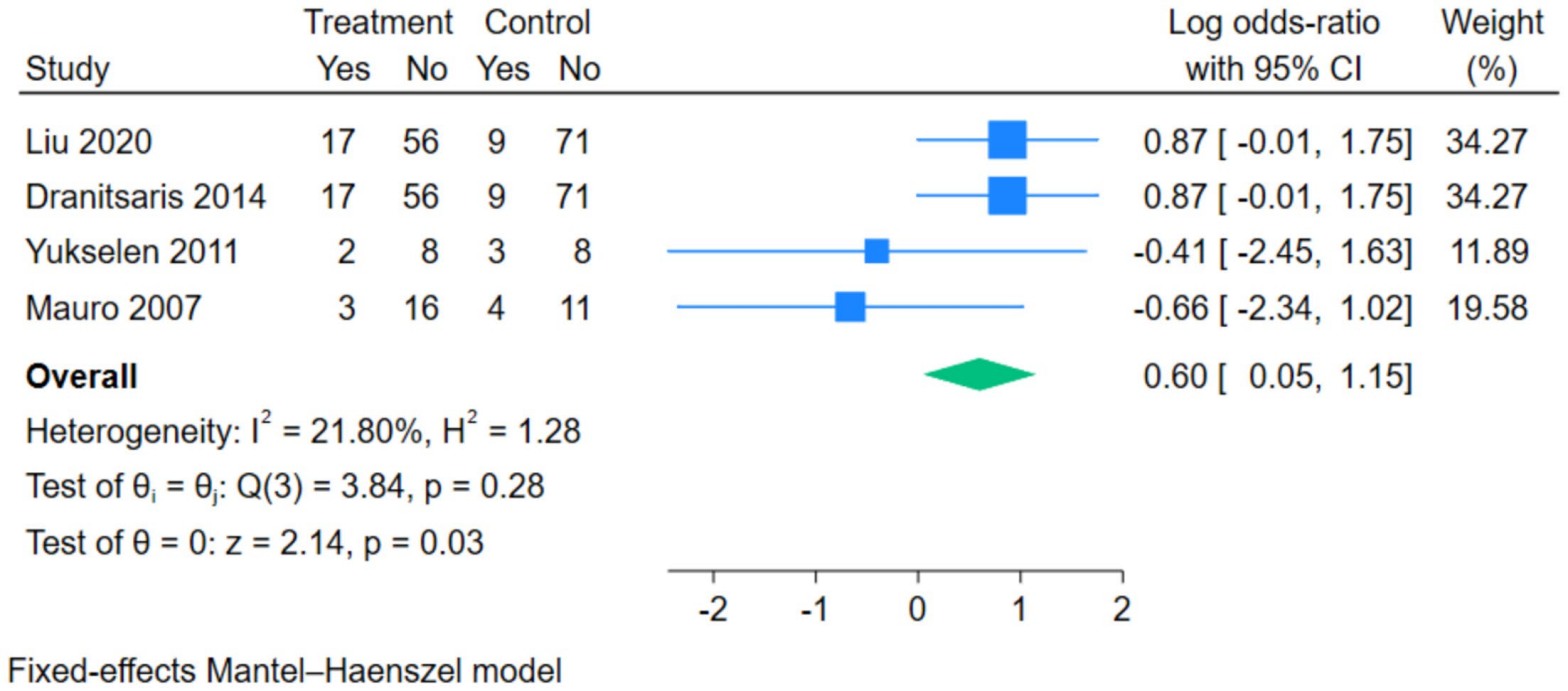
Forest plot of the incidence of adverse reactions.

The forest plot of adverse reaction incidence rates showed that four studies examined the correlation between subcutaneous immunotherapy (SCIT) and sublingual immunotherapy (SLIT) in the treatment of allergic rhinitis. The Q-test and I^2^ test indicated no heterogeneity among the studies (Chi^2^ = 3.84, df = 3, *p* = 0.28, I^2^ = 21.80%). Therefore, the fixed-effects model was used to calculate the log-odds ratio (log OR). The results showed a statistically significant difference in the risk of adverse reactions between patients treated with SCIT and those treated with SLIT (pooled log OR = 0.60, 95% CI [0.05, 1.15], Z = 2.14, *p* = 0.03) (Figure 4).

### Publication bias

To test for publication bias, funnel plots were drawn.

The funnel plot of the treatment interruption rate showed an asymmetric distribution, suggesting potential publication bias. Furthermore, Egger’s test (random-effects model, REML method) was conducted. The results showed that Z = 0.43 and *p* = 0.664, which is greater than 0.05, suggesting that no publication bias was identified.

**Figure 5:**
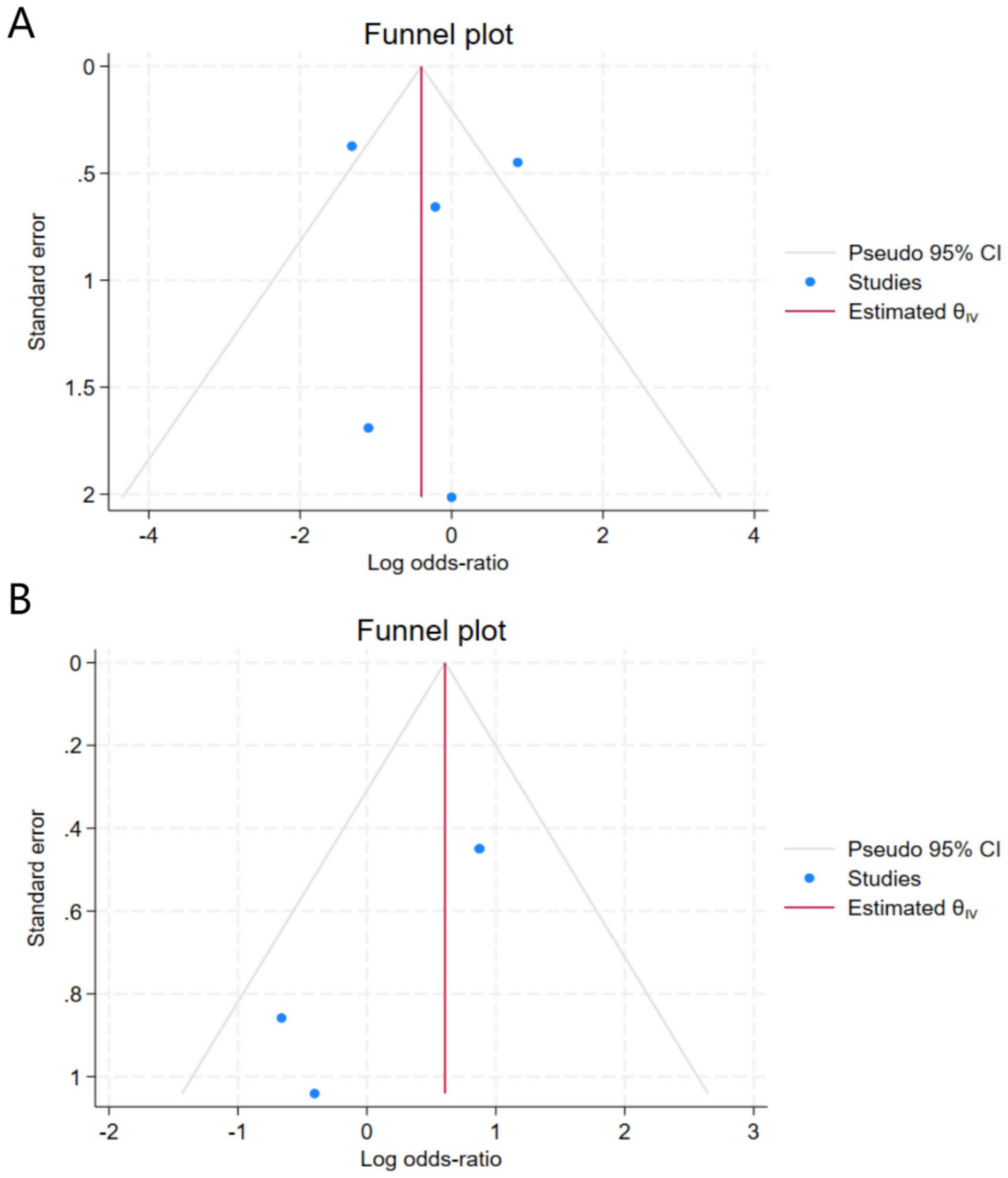
Funnel plots **(A)** treatment discontinuation rate and **(B)** adverse reaction incidence rate.

The funnel plot of the incidence of adverse reactions presented an asymmetric distribution, hinting at possible publication bias. Furthermore, Egger’s test (fixed-effects model, inverse-variance method) was performed. The results showed that Z = -1.87 and *p* = 0.061, which is greater than 0.05, suggesting that no publication bias was found (Figure 5).

### Sensitivity analysis

Sensitivity analysis was conducted by excluding each of the studies one at a time for the two primary outcomes. For the treatment discontinuation rate, after excluding each of the five included studies in turn, the pooled OR direction remained unchanged, and the I^2^ value ranged from 0 to 56.2%, indicating that the conclusion of the meta-analysis for this outcome was relatively robust. For the adverse event rate, after excluding each of the four included studies in turn, the pooled OR direction and statistical significance remained unchanged, and the I^2^ value was consistently 0–25.8%, suggesting a high-level of robustness for the conclusion regarding this outcome.

### Sub-group analysis

#### Treatment discontinuation rate

**Figure 6:**
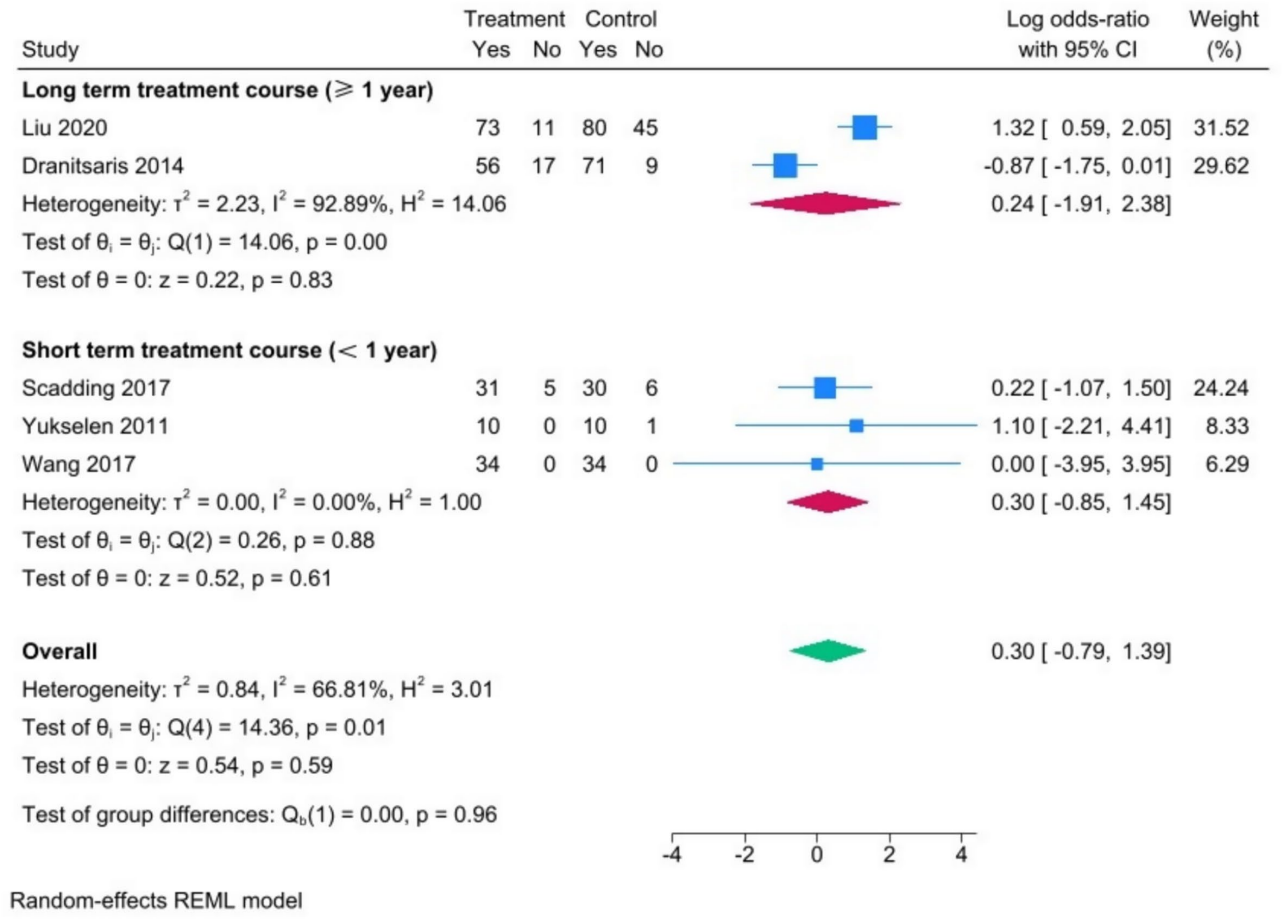
Forest plot of subgroup analysis of the recurrence rate.

In the subgroup analysis for long-term treatment course (≥1 year, defined as continuous treatment for more than 12 months), heterogeneity was observed (I^2^ = 85.38%, H^2^ = 6.84), extremely high heterogeneity was observed (Chi^2^ = 14.06, df = 1, *p* = 0.00, I^2^ = 92.89%, H^2^ = 14.06), and the pooled log odds ratio was 0.24 (95% CI: [−1.91, 2.38]). The difference was not statistically significant (Z = 0.22, *p* = 0.83). In the subgroup analysis of Liu (10), the log odds ratio was 1.32 (95% CI: [0.59, 2.05]). In the subgroup analysis of Dranitsaris (11), the log odds ratio was −0.87 (95% CI: [−1.75, 0.01]). In the subgroup analysis stratified by treatment duration (long-term ≥1 year vs. short-term <1 year), significant heterogeneity was observed among studies in the long-term treatment group (I^2^ = 85.38%, H^2^ = 6.84), while no heterogeneity was found in the short-term subgroup I^2^ = 0%). The pooled log odds ratio was 0.30 (95% CI: [−0.85, 1.45]). The difference was not statistically significant (Z = 0.52, *p* = 0.61). In the subgroup analysis of Scadding (12), Yukselen (13), and Wang (14), the log odds ratios were 0.22 (95% CI: [−1.07, 1.50]), 1.10 (95% CI: [−2.21, 4.41]), and 0.00 (95% CI: [−3.95, 3.95]), respectively (Figure 6).

### GRADE evidence quality assessment

The GRADE grading standards were used to assess the quality of the evidence for the primary and secondary outcomes in this study. The results showed that the evidence quality for the treatment discontinuation rate was moderate, with the downgrading reason being moderate heterogeneity among studies; and the evidence quality for the adverse event rate was moderate, with the downgrading attributed to the small sample size of the included studies. No upgrades were applied across all outcomes, and the final evidence quality grade ranged from moderate to low.

## Discussion

In this study, the efficacy and safety of SCIT and SLIT for patients with allergic rhinitis were systematically evaluated using meta-analysis. Based on a combined analysis of six randomized controlled trials, the main result showed that there was no significant difference between SCIT and SLIT in terms of treatment discontinuation and adverse event rates.

In terms of treatment compliance, the combined results of six studies showed no significant difference in the treatment interruption rate between SCIT and SLIT. While SCIT requires regular in-hospital injections, SLIT can be self-administered at home. This study did not find a difference in the interruption risk between the two treatments. This can be explained by the following factors: First, the efficacy of SCIT has enhanced patients’ willingness to adhere to treatment, which helps to mitigate the inconvenience of receiving injections (15–17); second, the included studies have standardized follow-up management, ensuring that clinicians provide similar medication guidance and adherence monitoring for both groups. This reduces fluctuations in discontinuation rates caused by differences in management practices; third, the baseline characteristics of patients (such as AR severity and comorbidity status) in each study are relatively balanced, and there is no significant difference in disease severity between the two treatment groups, avoiding outcome deviations caused by baseline disparities.

In the safety assessment, the combined analysis of five studies found no significant difference in the incidence of adverse reactions between the two treatment methods. This result can be attributed to the following mechanisms: First, although SCIT is an invasive administration method, the optimization of modern formulation technology (such as standardized allergen extracts and gradual dose escalation during the induction phase) has significantly reduced the risk of serious adverse events (17, 18). While SLIT is non-invasive, mild local reactions (such as sublingual mucosal irritation) may occur due to the relatively high allergen dose in some studies, and the differing spectra of adverse reactions between the two therapies counterbalance each other, leading to no significant difference in the overall incidence of adverse reactions. Second, the adverse events of both therapies are mostly mild to moderate: SCIT mainly results in localized reactions at the injection site (such as redness and swelling), while SLIT is dominated by local oral irritation. Neither therapy leads to a significant increase in discontinuation rates due to intolerable reactions, which also contribute to their comparable overall safety profiles. This result provides an important safety foundation for clinical decision-making, indicating that clinicians do not need to overemphasize the safety differences between the two therapies when making their choices.

In this study, the heterogeneity of the treatment discontinuation rate and the incidence of adverse reactions was relatively low. A preliminary analysis identified the main sources of heterogeneity as follows: first, differences in the baseline characteristics of the included patients, such as allergen types (mainly dust mites and pollen), disease duration (3 months to 5 years), and age distribution (a mix of children and adults); second, differences in treatment regimens, such as allergen dose (50,000 SQ to 100,000 SQ), administration frequency (once a week to once a month), and treatment course (6 months to 3 years); third, inconsistent timing of outcome assessments (3 months, 6 months, and 1 year after treatment). This study did not conduct a stratified analysis based on the above key factors; therefore, it was unable to clarify the contribution of each factor to the heterogeneity, which is an important limitation of this study. Some studies evaluated short-term effects, and others focused on long-term outcomes (18, 19). After excluding any one study one by one, the direction of the combined effect size did not change; but after excluding the Proctor 2020 study, the direction of the effect size of the treatment interruption rate changed, suggesting that the reliability of the conclusion of this indicator is relatively insufficient, and clinical interpretation should be cautious. The funnel plots showed that the four outcome indicators exhibited asymmetric distributions, but the results of Egger’s test showed that all *p*-values were >0.05, and no obvious publication bias was found. This contradiction may be due to the limited number of included studies (6), resulting in a bias in the visual judgment of the funnel plot (19, 21, 22). Overall, the risk of publication bias in this study is low, and the results are highly credible.

This study has many advantages: the retrieval strategy comprehensively covers the five major mainstream databases, namely PubMed, Medline, Web of Science, Cochrane Library, and EMBASE. The time span extends to August 2025, effectively ensuring the timeliness and comprehensiveness of the included studies and reducing the omission bias. In strict accordance with systematic review specifications, two researchers independently carried out the literature screening, data extraction, and quality evaluation, and cross-checking was conducted to reduce human bias. For the included randomized controlled trials, the Cochrane RoB-2 tool was uniformly used for quality assessment. All included studies were of medium- to high-quality, with no definite high-risk biases, ensuring the reliability of the data. At the same time, subgroup analysis, sensitivity analysis, and publication bias test were carried out on the core outcome indicators to comprehensively verify the robustness of the results and improve the credibility of the conclusions.

First, this study included only 6 RCTs with a total sample size of 588 cases. The limited number of included studies and small sample size led to insufficient statistical power. It should be noted that the subgroup analyses in this study (e.g., stratified by treatment course or allergen type) were exploratory, given the small number of included studies per subgroup (1–2 studies). The limited sample size within subgroups may result in insufficient statistical power to detect potential differences, so subgroup results should not be used as the sole basis for clinical decision-making. Clinicians should combine the overall results, patient characteristics (e.g., allergen type and treatment accessibility), and clinical experience when selecting therapies. We will expand the scope of retrieval to include more studies in the follow-up. Second, the heterogeneity of treatment regimens was not completely eliminated, and differences in allergen types, doses, and courses in different studies may affect the consistency of the results. Third, outcome measures were incomplete: the current analysis focused only on primary outcomes (treatment discontinuation rate, adverse event rate) due to limitations in data availability. Clinically relevant outcomes such as quality of life (e.g., Rhinoconjunctivitis Quality of Life Questionnaire [RQLQ] scores) and recurrence rate were not included—only one included study mentioned QoL without complete extractable data, and no studies systematically reported the recurrence rate (e.g., symptom recurrence within 1 year after treatment completion). This limits a comprehensive evaluation of the clinical value of two therapies beyond adherence and safety. Fourth, long-term prognostic indicators, such as recurrence rates, were not analyzed; therefore, the long-term clinical benefits of the two treatment methods could not be fully reflected. Fifth, confounding variables, such as the doctor’s experience and patients’ comorbidities, were not fully controlled/ The heterogeneity of treatment regimens was not completely eliminated, and differences in allergen types, doses, and courses in different studies may affect the consistency of the results.

Future studies can be carried out from multiple dimensions to improve the evidence base: first, researchers should expand the scope of literature retrieval, supplement the search in both Chinese and English core databases, and include as many RCTs comparing SCIT and SLIT as possible to improve the statistical power of the meta-analysis; second, multi-center, large-sample randomized controlled trials are needed to unify the protocol parameters and outcome indicator evaluation criteria of AIT to provide higher-level evidence-based foundation for clinical practice; third, the follow-up time should be extended to focus on the long-term efficacy persistence and the recurrence risk of patients undergoing SCIT and SLIT to provide data support for the development of clinical treatment cycles; fourth, subgroup analysis can be carried out for different allergen types and age stratifications to clarify the differences in the applicable populations for the two therapies and assist in individualized treatment recommendations (23). At the same time, patient-reported outcome indicators, such as quality of life and treatment satisfaction, should be combined to comprehensively evaluate the clinical value of the therapies. In addition, pharmacoeconomic studies should be carried out to compare the cost-effectiveness ratio of the two therapies to provide valuable insights for the optimal allocation of medical resources.

In conclusion, SCIT and SLIT exhibited comparable treatment adherence (discontinuation rates) in patients with allergic rhinitis; the incidence of adverse events in SCIT was significantly higher than in SLIT, and SLIT had greater safety advantages. Clinical decisions should consider individual patient factors, including treatment convenience and monitoring conditions.

## Data Availability

The original contributions presented in the study are included in the article/supplementary material, further inquiries can be directed to the corresponding author.
